# Qualitative, Quantitative, Cytotoxic, Free Radical Scavenging, and Antimicrobial Characteristics of *Hypericum lanuginosum* from Palestine

**DOI:** 10.3390/molecules27144574

**Published:** 2022-07-18

**Authors:** Nidal Jaradat

**Affiliations:** Department of Pharmacy, Faculty of Medicine and Health Sciences, An-Najah National University, Nablus P.O. Box 7, Palestine; nidaljaradat@najah.edu

**Keywords:** *Hypericum lanuginosum*, phytochemicals, antibacterial, antifungal, antiproliferative, oxidative stress

## Abstract

*Hypericum lanuginosum* is one of the traditional medicinal plants that grows in the arid area of the Al-Naqab desert in Palestine and is used by Bedouins to heal various communicable and non-communicable illnesses. The purpose of this investigation was to estimate the total phenolic, flavonoid, and tannin contents of aqueous, methanol, acetone, and hexane *H. lanuginosum* extracts and evaluate their cytotoxic, anti-oxidative, and antimicrobial properties. Qualitative phytochemical tests were used to identify the major phytochemical classes in *H. lanuginosum* extracts, while total phenol, flavonoid, and tannin contents were determined using Folin–Ciocalteu, aluminum chloride, and vanillin assays, respectively. Moreover, a microdilution test was employed to estimate the antimicrobial activity of *H. lanuginosum* extracts against several microbial species. At the same time, the cytotoxic and free radical scavenging effects were evaluated using 3-(4,5-dimethylthiazol-2-yl)-5-(3-carboxymethoxyphenyl)-2-(4-sulfophenyl)-2H-tetrazolium (MTS) and 2, 2-diphenyl-1-picryl-hydrazyl-hydrate (DPPH) assays, respectively. Quantitative examinations showed that the highest amounts of phenols, flavonoids, and tannins were noticed in the *H. lanuginosum* aqueous extract. Moreover, *H. lanuginosum* aqueous extract showed potent activity against methicillin-resistant *Staphylococcus aureus* even more than Amoxicillin and Ofloxacin antibiotics, with Minimum Inhibitory Concentrations (MICs) of 0.78 ± 0.01, 0, and 1.56 ± 0.03 µg/mL, respectively. Additionally, the aqueous extract exhibited the highest activity against *Candida albicans* and *Epidermatophyton floccosum* pathogens, with MIC values of 0.78 ± 0.01 µg/mL. Actually, the aqueous extract showed more potent antimold activity than Ketoconazole against *E. floccosum* with MICs of 0.78 ± 0.01 and 1.56 ± 0.02 µg/mL, respectively. Furthermore, all *H. lanuginosum* extracts showed potential cytotoxic effects against breast cancer (MCF-7), hepatocellular carcinoma (Hep 3B and Hep G2), and cervical adenocarcinoma (HeLa) tumor cell lines. In addition, the highest free radical scavenging activity was demonstrated by *H. lanuginosum* aqueous extract compared with Trolox with IC_50_ doses of 6.16 ± 0.75 and 2.23 ± 0.57 µg/mL, respectively. Studying *H. lanuginosum* aqueous extract could lead to the development of new treatments for diseases such as antibiotic-resistant microbes and cancer, as well as for oxidative stress-related disorders such as oxidative stress. *H. lanuginosum* aqueous extract may help in the design of novel natural preservatives and therapeutic agents.

## 1. Introduction

Not so many years ago, herbal remedies were the only effective weapon in the armory of physicians. Even today, naturally occurring remedies are of paramount importance in the fight against infectious and non-infectious illnesses [1]. In daily life, human beings are exposed to various types of pathogens such as bacteria, viruses, fungi, and parasites. In fact, many types of microbial infections harm human bodies, and in some instances, they can be fatal [2]. Therefore, pharmaceutical companies are working hard to develop different types of antimicrobial agents to overcome this problem [3].

Excessive oxidative stress triggers the formation of reactive oxygen species, which have been linked to several other recent fatal diseases, including diabetes, cancer, neurodegenerative, and cardiovascular diseases [4]. The genomic integrity of the cell is maintained by a balance between the levels of pro-oxidants and antioxidants [5]. If this balance is disrupted, the host immunity is modulated and the normal cellular signaling pathways are affected, leading to uncontrolled proliferation of the cells and ending in cancer, and macrophage polarization leads to the formation of atherogenic plaques [5,6].

Cancer is a broad term that describes the disease that results when cellular changes cause the uncontrolled growth and division of cells. Some types of cancer cause rapid cell growth, while others cause cells to grow and divide at a slower rate. Certain forms of cancer result in visible growths called tumors, while others, such as leukemia, do not [7]. Chemotherapy, hormonal therapy, immunotherapy, precision medicine, radiation therapy, stem cell transplant therapy, surgery, and targeted therapies are the most commonly used methods for the treatment of cancer disease [8].

The genus of the *Hypericum* plant compromises more than 460 species that are wildly and broadly distributed in all world regions [9]. Many researchers have documented several pharmacological properties of various *Hypericum* species, including antidepressant, antiproliferative, antimicrobial, antioxidant, anti-inflammatory, and neuroprotective effects [10]. In traditional medicine, different *Hypericum* species have been used in folk medicine in sore furuncle, bruises, wounds, stomatitis, sore throat, strangury, dysentery, and hepatitis remedies. Additionally, they are used as a treatment for various types of cancer, including nasopharyngeal, laryngeal, tonsil, lung, bladder, and prostatic carcinomas [10,11].

*Hypericum lanuginosum* Lam. is a perennial herb plant in the Hypericaceae family that can reach 0.8 m in height, with few green and terete stems. Its leaves are sub-glabrous on both surfaces, with whitish and thick veins. *H. lanuginosum* is found in the desert of Sinai (Egypt), Southern Turkey, Western parts of Syria, and arid areas of Palestine and Jordan [12].

Ebru reported that 41 molecules were recognized in the essential oil of *H. lanuginosum* aerial parts from Turkey, with spathulenol, caryophyllene oxide, α-pinene, and undecane [9].

In addition, Mahomoodallya et al. investigated the chemical components of *H. lanuginosum* organic extracts and detected quinic acid, flavonoid, acylquinic acids, and phenolic acids by the LC-HRMS technique [13]. Moreover, Odabas et al. reported that *H. lanuginosum* leaves methanol extract was abundant in quercitrin and neochlorogenic acid [14].

So far, only two phytochemical investigations and one biological study have been conducted on this species [9,13], while its antimicrobial and cytotoxic properties have not been investigated yet as far as the recent literature is concerned.

Therefore, the present study aims to conduct a qualitative and quantitative analysis of the cytotoxic, antibacterial, and antifungal characteristics of the *n*-hexane, acetone, methanol, and water extracts of the *H. lanuginosum* plant from Palestine.

## 2. Results

### 2.1. Qualitative Assessments

The conducted analytical tests identified the presence of different phytochemical classes of secondary and primary metabolites in the four solvent extracts of the *H. lanuginosum* plant. The results of the identification tests showed that the methanolic extract was the richest in bioactive molecules and contained seven metabolic phytochemical classes, including flavonoids, tannins, phenols, cardiac glycosides, monosaccharides, reducing sugars, and steroids (Table 1), followed by the aqueous extract, which contained flavonoids, tannins, phenols, proteins, cardiac glycosides, and steroids. On the other hand, flavonoids, tannins, and phenols were found in the acetone extract, and steroids were found in the *H. lanuginosum* hexane extract.

#### 2.1.1. Estimation of Total Phenol Content

Quantitative analysis of phenol content in the four solvent extracts of the *H. lanuginosum* plant was performed using gallic acid as a reference compound, and the results are expressed as mg gallic acid equivalent per gram of dry weight for each extract (mg GAE/g DW). However, the total phenol contents were calculated from the following equation:

y = 0.0056x + 0.0495 R² = 0.981,

where y is the absorbance at 765 nm and x is the total phenol content of the plant extracts, which was obtained from the calibration curve that is given in the Supplementary Materials (Figure S1).

#### 2.1.2. Assessment of Total Tannin Content

A quantitative analysis of tannin content in the four solvent extracts of *H. lanuginosum* was performed using catechin as a reference compound and expressed as catechin equivalent per gram of dry weight for each of the plant extracts (mg CAE/g DW). According to the standard catechin calibration curve (Figure S2), the following equation was utilized to estimate the total tannin content in the four solvent plant extracts.

y = 0.0009x + 0.011, R^2^ = 0.9956,

where y is the absorbance at 510 nm and x is the total tannin content in each plant extract.

#### 2.1.3. Flavonoid Content 

Total flavonoids content was calculated from the calibration curve of quercetin and expressed as milligram of Quercitin Equivalent per gram of extract (mg QUE/g extract).

From the quercetin’s calibration curve (Figure S3), the following equation was obtained to determine the total tannin content of the four plant extracts:

y = 0.0004x + 0.0011, R^2^ = 0.995.

The total contents of phenol, tannin, and flavonoid quantitative tests results of the hexane, acetone, methanol, and aqueous extracts of the *H. lanuginosum* plant are summarized in Table 2.

### 2.2. Antimicrobial Activity

*H. lanuginosum* hexane, aqueous, acetone, and methanol extracts were tested for antimicrobial activity using the microdilution technique against *C*. *albicans, E*. *floccosum*, *E*. *coli*, *P*. *aeruginosa*, *S*. *sonnie*, *E*. *faecium*, *S*. *aureus*, and MRSA. Table 3 shows that most of the screened extracts have potential antimicrobial activity compared with the used positive control antibiotics in this study.

### 2.3. Cytotoxic Activity

In the present investigation, MTS was employed to estimate the cytotoxic effects of methanol, aqueous, hexane, and acetone extracts of *H. lanuginosum* on the cell proliferation of breast cancer (MCF-7), hepatocellular carcinoma (Hep 3B and Hep G2), and cervical adenocarcinoma (HeLa). Cells were exposed for 24 h to increasing concentrations of the tested extracts (0, 0.05, 0.1, 0.3, 0, 5, 1, and 2 µg/mL).

**Figure 1:**
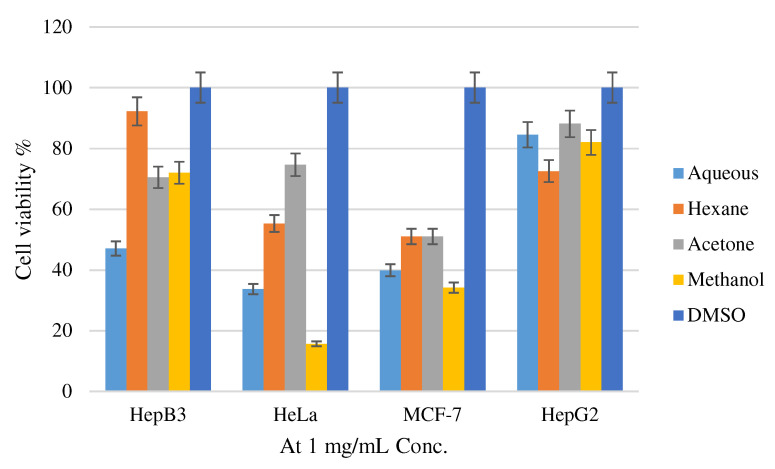
Cell viability % of *H. lanuginosum* methanol, aqueous, hexane, and acetone extracts.

The results of the inhibitory effect of methanol, aqueous, hexane, and acetone extracts of *H. lanuginosum* in addition to Doxorubicin on cancer cell lines viability in the present study ranged from 15.66–88.1% at one µg/mL as shown in Figure 1, and the IC_50_ values are demonstrated in Table 4.

### 2.4. Free Radical Scavenging Activity

Figure S4 depicts the free radical scavenging activity of various *H. lanuginosum* extracts and the standard compound Trolox. The results revealed that all the tested samples had free radical scavenging effects, but among the extractives, aqueous extract possessed the highest free radical scavenging influence. However, the antioxidant activity IC_50_ values of *H. lanuginosum* four extracts and Trolox are listed in Table 5, where the higher antioxidant effect is indicated by a lower IC_50_ value.

## 3. Discussion

Plants and their extracts have been utilized for hundreds of years in therapeutic remedies, food preservatives, and cosmetics. They have also been used for the treatment of various animal and human diseases, including infectious, systematic, and inflammatory diseases since the Hippocrates era (460–377 BC) [15].

### 3.1. Qualitative Phytochemical Screening

The identification tests of *H. lanuginosum* methanol, acetone, and water extracts detected the presence of therapeutically active secondary metabolic phytochemical groups such as cardiac glycosides, phenols, steroids, tannins, and flavonoids. A steroidal phytochemical group was the only detected phytochemical group in *H. lanuginosum* hexane extract.

#### Total Phenol, Flavonoid, and Tannin Contents

Plant secondary metabolites with an aromatic ring and at least one hydroxyl group are known as phenolic substances, such as flavonoids, simple phenols, and tannins. More than 8000 naturally occurring phenolic compounds from plants have been identified [16,17]. Studies on flavonoids and other phenolic compounds from medicinal plant species have grown significantly over the past several decades because of their numerous health advantages for humans [18]. Phenolic constituents have been reported for their effectiveness as anticancer, antibacterial, and antioxidant agents [19].

The results in the current study showed that the highest extraction yields were exhibited by polar solvents such as water, methanol, and acetone, and the lowest yield was observed in the non-polar hexane solvent. However, the yields of *H. lanuginosum* extracts were found to be 4.3 ± 0.22%, 2.2 ± 0.13%, 1.8 ± 0.03%, and 1.2 ± 0.02%, respectively. In the current investigation, total phenolic compounds were found in the acetone, methanol, and aqueous extracts of *H. lanuginosum*. The highest total phenol content was found in the aqueous (92.41 ± 1.54 mg GAE/g of plant extract) and methanol (32.82 ± 0.81 mg GAE/g plant extract) extracts, followed by the acetone extract (1.41 ± 0.25 mg of GAE/g of plant extract), whereas phenolic compounds were absent in the hexane extract. At the same time, the highest amounts of flavonoid content were estimated in the aqueous (76.7 ± 2.01 mg QUE/g of plant extract) and methanolic (28.21 ± 1.02 mg QUE/g of plant extract) *H. lanuginosum* extracts. In addition, aqueous *H. lanuginosum* extract contains the highest contents of tannins (15.11 ± 0.71 mg CAE/g of plant extract).

Only one study was found in the literature by Mahomoodally et al. that screened total phenols, tannins, and flavonoid contents in the *H. lanuginosum* from Turkey, which reported that the aqueous extract had the highest total phenol (168.56 mg GAE/g extract) and flavonoid (53.22 mg RE/g extract) contents among other investigated ethyl acetate and methanolic extracts. The methanol extract was in second place with a total phenol content of 72.03 mg GAE/g extract and a total flavonoid content of 30.96 mg RE/g extract [13]. However, to the best of our knowledge, no previous studies investigated total tannin content.

In fact, the current study outcomes agreed with the quantitative test results of Mahomoodally et al. As mentioned above, the highest total phenols and flavonoid contents were the abundant amounts in the aqueous *H. lanuginosum* extract.

As it is well known, herbs within the same species show significant variation in their chemical components based on their geographical locations associated with environmental interactions, including the type of soil, climate, rainfall changes, and growth stages [20].

### 3.2. Antimicrobial Activity

Current antimicrobial drugs have been used for the treatment of human and animal infections for less than a century. The development of drug-resistant pathogens has occurred rapidly, while the emergence of multi-drug resistant strains has increased exponentially in recent years [21].

The microdilution assay was used to evaluate the antimicrobial activity of the hexane, aqueous, acetone, and methanol solvent extracts of *H. lanuginosum*. The results revealed that the aqueous extract of *H. lanuginosum* exhibited the greatest antibacterial activity against the *E. coli*, *P. aeruginosa*, *S. sonnie*, *E. faecium*, *S. aureus*, and MRSA strains with MIC values of 3.13 ± 0.04, 1.56 ± 0.02, 1.56 ± 0.01, 0.78 ± 0.01, 0.78 ± 0.01, and 0.78 ± 0.01 µg/mL followed by the methanol extract with MIC value of 6.25 ± 0.88, 3.13 ± 0.01, 12.5 ± 0.41, 3.13 ± 0.01, 1.56 ± 0.21, and 3.13 ± 0.05 µg/mL, respectively. The hexane extract did not affect the growth of all the tested bacterial strains except against *S. aureus* (MIC = 3.13 ± 0.12 µg/mL). In comparison, the acetone extract inhibited the growth of most of the tested bacterial strains but with weak activities.

The aqueous and methanol extracts showed the highest anticandida and antimold activities against *C*. *albicans* and *E. floccosum* with MIC values of 0.78 ± 0.01 and 1.56 ± 0.02 µg/mL, respectively. Finally, the aqueous and methanol extracts exhibited the highest antibacterial and antifungal activities compared with Amoxicillin, Ofloxacin, and Ketoconazole antibiotics, which were used as a positive control. Against MRSA, the *H. lanuginosum* aqueous extract showed potent activity, even more than Amoxicillin and Ofloxacin antibiotics, with MICs of 0.78 ± 0.01, 0, and 1.56 ± 0.03 µg/mL, respectively. That could be seen as a good result since MRSA is one of the most dangerous hospital pathogens and affects many countries.

Concerning the antifungal activity of *H. lanuginosum* extracts, the results indicated that the aqueous extract exhibited the highest activity against *C. albicans* and *E. floccosum* pathogens, with MIC values of 0.78 ± 0.01 µg/mL followed by the methanol extract (MICs of 1.56 ± 0.02 µg/mL). Actually, the aqueous extract showed more potent antimold activity than Ketoconazole against *E. floccosum* with MICs of 0.78 ± 0.01 and 1.56 ± 0.02 µg/mL, respectively.

The antimicrobial activity of *H. lanuginosum* has not been investigated yet as far as the literature is concerned, and our study is the first to investigate this important biological activity.

As presented in the *H. lanuginosum* quantitative tests, the aqueous and methanolic extracts have the highest content of phenols, flavonoids, and tannins, while the qualitative tests show that these extracts are the richest ones with secondary metabolites. This shows that there is a positive correlation between the chemical constituents of the aqueous and methanolic extracts of the *H. lanuginosum* plant and antimicrobial activity. Several studies suggest that phytochemical classes such as tannins, flavonoids, and phenols have been known to be biologically active and thus responsible for the antimicrobial activities of plants [22,23].

### 3.3. Cytotoxic Effects

The term ”cancer” refers to a collection of over 100 diseases that can affect any organ of the body. These diseases are usually typically deadly and impose a colossal burden on society and the health care system [24]. Along with conventional medicine, some people choose other methods of cancer treatment such as complementary and alternative medicine or traditional medicine, particularly medicinal plants [25].

By employing the MTS colorimetric assay, the cell proliferation of MCF-7, Hep 3B, Hep G2, HeLa, and Hek293t cell lines were used to estimate *H. lanuginosum* methanol, aqueous, hexane, and acetone extracts’ cytotoxic effects. Cells were exposed for 24 h to increasing concentrations of the tested extracts (0, 0.05, 0.1, 0.3, 0, 5, 1, and 2 µg/mL).

Results indicated that all the tested plant extracts exerted cytotoxic activity against all the cancer cell lines utilized in this investigation. However, the highest cytotoxic effect against the Hep 3B cell line was noticed by the *H. lanuginosum* aqueous extract, with an IC_50_ value of 46.9 ± 1.01 µg/mL. The methanol extract had the highest cytotoxic effect against HeLa and MCF7 cells, with IC_50_ doses of 16.07 ± 0.25 and 33.92 ± 0.91 µg/mL, respectively. In addition, the highest cytotoxic effect against the Hep G2 cancer cell line was noticed by *H. lanuginosum* hexane extract followed by aqueous extract, with IC_50_ values of 73 ± 0.57 and 85.02 ± 0.52 µg/mL, respectively. All the obtained results were compared with Doxorubicin, the potent chemotherapeutic agent. The cytotoxic effects of the *H. lanuginosum* extracts were also evaluated against the normal cell line (Hek293t), and the results showed that the evaluated *H. lanuginosum* methanol, aqueous, hexane, and acetone extracts are not toxic, and the IC_50_ values were 331.87 ± 3.12, 190.16 ± 2.67, 176.02 ± 1.99 and 288.96 ± 1.55 µg/mL, respectively, compared with Doxorubicin, which was highly cytotoxic (IC_50_ = 0.58 ± 0.07 µg/mL).

Based on these facts, *H. lanuginosum* methanol, aqueous, hexane, and acetone extracts contain several phytochemical molecules, including flavonoids, phenols, and tannins, which have cytotoxic effects as previously reported in the literature. In addition, many research teams consider the synergic effect of these compounds important, so flavonoids, phenols, and tannins-rich plant extracts show different potential cytotoxic effects on various cancer cell lines [23].

### 3.4. DPPH-Free Radical Scavenging Activity

The DPPH-free radical test is extensively used to assess a compound’s or extract’s potential to act as free radical scavengers and hydrogen providers. It is a quick, easy, and low-cost approach for assessing antioxidant capacity [26,27].

The current experiment revealed that *H. lanuginosum* methanol, aqueous, hexane, and acetone extracts scavenged DPPH free radicals in a concentration-dependent manner. In fact, the aqueous extract exhibited the most potent free radical scavenging activity, followed by the methanolic extract with IC_50_ dosages of 6.16 ± 0.75 and 52.48 ± 1.31 µg/mL, respectively, in comparison to the positive control Trolox (IC_50_ = 2.23 ± 0.05 µg/mL), which is a potent antioxidant medication. Meanwhile, the acetone extract was a weak free radical scavenger, and the hexane extract was almost inactive.

The study by Mahomoodally et al. showed that the methanol and aqueous extracts of the *H. lanuginosum* plant collected from Turkey had an antioxidant activity with IC_50_ doses of 105.93 ± 2.07 and 354.60 ± 7.29 mg TE/g, respectively [13].

All these outcomes may be attributed to the presence of a variety of polyphenols in the *H. lanuginosum* plant polar extracts. As reported in the literature, the *H. lanuginosum* polar extract contains twenty-one phenolic compounds, including bioflavonoids, flavonoids, acylquinic acids, and phenolic acids, based on the chemical components of *H. lanuginosum* aqueous and methanol extracts that were identified [13].

The powerful antioxidant activity of these extracts seems to depend on the presence of carboxylic acid (COOH) in acylquinic and phenolic acids, as well as two hydroxyl (OH) groups in the ortho position of the benzene ring in flavonoids and bioflavonoids [28].

Advanced quantitative and qualitative tests using LC-MS, HPLC-DAD, or GC-MS techniques are required in order to identify the molecules responsible for the biological activities. Moreover, in vivo studies are needed to elucidate the potential of the *H. lanuginosum* aqueous and methanolic extracts as anticancer, antimicrobial, and antioxidant agents, and their possible mechanisms of action, in addition to the toxicological investigations needed to prove their safety.

## 4. Materials and Methods

### 4.1. Chemical Reagents

Methanol, *n*-hexane, and Acetone (Loba chemie, Mumbai, India) were used to prepare the solvent extracts. Dimethyl sulphoxide (DMSO; Riedel-de Haën, Seelze, Germany) and Mueller–Hinton broth (Himedia, Maharashtra, India) were used for the antimicrobial screening experiments.

Reagents used for the quantitative and qualitative assessments (ninhydrin solution, Benedict’s reagent, Millon’s reagent, magnesium ribbon, iodine, sulphuric acid, acetic acid, and Molish’s reagent) were obtained from Alfa Aesar (Lancashire, UK). NaHCO_3_, FeCl_3_, vanillin, HCl, gallic acid, quercetin, sodium nitrite, AlCl_3_, and chloroform were obtained from Sigma-Aldrich (Schnelldorf, Germany). Trolox was purchase from Sigma-Aldrich (St. Louis, MO, USA) was ((s)-6-hydroxy-2,5,7,8-tetramethyl chroman-2-carboxylic acid)), while DPPH reagent (2,2-diphenyl-1-1-picrylhydrazyl) was purchased from Sigma-Aldrich (Schnelldorf, Germany). For the cytotoxicity test, RPMI-1640 (Roswell Park Memorial Institute) medium was brought from Sigma-Norwich (Norfolk, UK). Pen-Strep solution, which consists of streptomycin and penicillin in concentrations of 10 mg/mL and 10,000 units/mL, respectively, was brought from BI (New Delhi, India), the L-glutamine solution was brought from Sigma (Welwyn Garden, UK), and the MTS reagent was purchased from Promega (Madison, WI, USA).

### 4.2. Instruments

A UV-visible spectrophotometer (Jenway 7315, Staffordshire, UK), balance (Radw, Gmina Górzyca, Poland), filter paper (Whatman no. 1, Marlborough, MA, USA), sonicator (MRC, 2014-207, Banbury, UK), water bath (LabTech, 2011051806, Namyangju, Korea), incubator (nüve, 06-3376, Ankara, Turkey), vortex (Heidolph, 090626691, Schwabach, Germany), autoclave (MRC, A13182, Banbury, UK), shaker incubator apparatus (Memmert, Büchenbach, Germany), lyophilizer (Mill-Rock Technology-BT85, Shanghai, China), rotary-evaporator (Heidolph, VV2000, Schwabach, Germany), grinder (Molineux I, Paris, France), microplate reader (Agilent BioTek, Winooski, VT, USA), and micro-broth plate from (Greiner/Bio-One, Ocala, FL, USA) were used in this study.

### 4.3. Plant Material

The aerial parts, including leaves and stems, of the *H. lanuginosum* plant were collected in May 2021 from the southern regions of the Hebron governorate of Palestine. Botanical determination was carried out in the Herbal Products Laboratory at An-Najah National University by a pharmacognosist, Dr. Nidal Jaradat. In the same laboratory, the plant sample was deposited under the voucher specimen code (Pharm-PCT-1239). The fresh parts were washed many times using tap water to remove soil and dust contamination and then washed again using distilled water. The clean parts were dried in the shade at 24 ± 3 °C and 55 ± 5 relative humidity for seven days. The prepared dry parts were then pulverized and kept in cotton bags for further use.

### 4.4. Preparation of H. Lanuginosum Extracts

The powdered plant material was sequentially extracted by the use of different solvents of increasing polarities to increase the extraction efficiency of phytochemicals, including the non-polar (hexane), polar aprotic (acetone), and polar protic solvents (methanol and water). In a special Pyrex^®^ glass bottle, 50 g of the grounded plant material was steeped in 0.5 L of four different solvents (water, methanol, acetone, and *n*-hexane) separately. Each bottle was placed in a sonicator device for 3 h at room temperature and then macerated for five days. Following this, each of the organic extracts was filtered and concentrated under a vacuum with a rotary evaporator. Finally, the aqueous extract was dried using a lyophilizer. The yields of the *H. lanuginosum* hexane, acetone, methanol, and aqueous extracts were found to be 1.2 ± 0.02%, 1.8 ± 0.03%, 2.2 ± 0.13%, and 4.3 ± 0.22%, respectively. The obtained extracts were kept at 5 °C for further analysis.

### 4.5. Phytochemical Qualitative Analysis

The *H. lanuginosum* extracts were qualitatively screened to detect the presence of secondary metabolic products comprising tannins, steroids, saponins, phenols, cardiac glycosides, alkaloids, and flavonoids. Qualitative screening for primary metabolites, including carbohydrates, protein, reducing sugars, and starch, was also performed. All the identification tests were performed according to the standard analytical assays as follows: 

A combination of Fehling’s solutions A and B in equal amounts was added to plant extracts. A red-colored precipitate indicates the presence of reducing sugars. Next, 2 mL of Molisch’s solution was mixed with plant extract. The presence of carbohydrates is shown by the appearance of a brown ring in the test tube’s interphase. A FeCl_3_ solution was added to the extract. Tannins were detected by their black or blue-green color. When 5 mL of distilled water was added to a crude plant extract and rapidly shaken, saponins were detected via the foam formation. Folin reagent was used to detect the presence of phenol, which appears in a dark blue color. Then, 2 mL of concentrated HCl was mixed with the plant extract. The mixture was then cooled, and 2 mL of KOH was added. A pink-red color indicated the presence of glycosides. Then, 2 mL of alkaloidal precipitating reagent mixed with plant extract, color change, or appearance of precipitate prove the presence of the alkaloids. Pieces of magnesium ribbon and concentrated HCl were mixed with crude plant extracts. After a few minutes, a pink-colored scarlet appeared that indicated the presence of flavonoids. The 2 mL of Millon’s reagent mixed with the plant extracts appeared as a white precipitate, which upon gentle heating turned into a red color, which indicated the presence of protein. A mixture of acetic acid glacial (2 mL) with two drops of 2% FeCl_3_ solution was added to the plant extract, and H_2_SO_4_ was concentrated. A brown ring was produced between the layers, which indicated the presence of cardiac glycosides. Then, 2 mL of chloroform and H_2_SO_4_ concentrated were mixed with the plant extracts. The lower chloroform layer produced a red color that indicated the presence of steroids [29].

### 4.6. Quantitative Chemical Analyses

#### 4.6.1. Total Phenol Content

The total phenol content was determined using the previous technique. [29]. In brief, a 1 mg/mL stock solution of gallic acid was made by dissolving 0.1 g gallic acid in 100 mL of methanol as a solvent in a volumetric flask, and then a series of concentrations (10, 20, 30, 40, 50, and 100 μg/mL) were prepared from this solution. The working mixture was prepared by mixing 0.5 mL of each methanolic solution, 2.5 mL of 10% Folin–Ciocalteu’s reagent diluted in water, and 2.5 mL of 7.5% NaHCO_3_. The samples were then stored in a thermostat at 30 °C for 1.5 h. The absorbance was measured utilizing a UV-Vis-spectrophotometer at a λ_max_ of 765 nm. The total phenol content was calculated using a calibration curve with gallic acid. The *H. lanuginosum* extracts were expressed as milligrams of gallic acid equivalents per gram of plant extract (mg GAE/g of plant extract).

#### 4.6.2. Total Tannin Content

Tannin content was estimated following the procedure of [30] utilizing catechin as a reference compound. Briefly, 100 µg/mL of aqueous stock solutions of methanolic extract were prepared then several dilutions (10, 30, 50, 70, and 100 μg/mL) were prepared. Thereafter, 1 mL from each dilution was added to 3 mL 4% vanillin (in methanol) solution and 1.5 mL of concentrated HCl. The absorbance was measured at 510 nm after 15 min of incubation. Total tannin content was expressed as milligrams of catechin equivalents per gram of plant extract (mg CAE/g of each plant extract).

#### 4.6.3. Total Flavonoid Content

The total flavonoid content of *H. lanuginosum* extracts was determined according to the procedure of [31]. It was calculated from the calibration curve of quercetin and expressed as milligrams of Quercitin Equivalent per gram of extract (mg QUE/g extract). Quercitin (100 mg) was dissolved in 10 mL of distilled water and diluted to 100 mL. Subsequently, the stock solution was diluted to provide a series of concentrations (10, 30, 50, 70, and 100 µg/mL). From each solution (0.5 mL) was mixed with 3 mL methanol, 0.2 mL of 10% AlCl_3_, 0.2 mL potassium acetate 1 M, and 5 mL distilled water and then incubated at room temperature for 30 min. Furthermore, absorbance was measured at 415 nm wavelength, and distilled water with methanol, 10% AlCl_3,_ and potassium acetate was used as a blank.

### 4.7. Antimicrobial Activities 

The microbial species utilized in the current investigation were obtained from the American Type Culture Collection (ATCC) and a selected methicillin-resistant *Staphylococcus aureus* (MRSA) strain, which was isolated in clinical settings in our region and exhibited multidrug resistance. The isolates included three Gram-positive strains: MRSA, *Enterococcus faecium* (ATCC 700221), and *Staphylococcus aureus* (ATCC 25923). The Gram-negative species *Shigella sonnie* (ATCC 25931), *Pseudomonas aeruginosa* (ATCC 27853), and *Escherichia coli* (ATCC 25922) were also used in the current investigation. Finally, the effect of the four extracts of *H. lanuginosum* on the fungal species *Candida albicans* (ATCC 90028) and *Epidermatophyton* *floccosum* (ATCC 10231) was also estimated. The extracts of *H. lanuginosum* were dissolved in DMSO to a concentration of 1 mg/mL. A broth microdilution test was used to determine Minimum Inhibitory Concentrations (MICs). Briefly, solutions were serially micro-diluted (2-fold) 10 times in sterile Mueller–Hinton broth. The dilution procedure was carried out in 96-well plates under aseptic conditions. In the micro-wells designated to test the antibacterial activity of plant extracts. A plant-free Mueller-Hinton broth was used as a positive control for microbial growth in micro-well number eleven. Plant-and microbe-free Mueller–Hinton broth was used as a negative control for microbial growth in micro-well number twelve. Micro-wells 1–11 were aseptically inoculated with the test microbes. All the inoculated plates were incubated at 35 °C. Regarding the fungal strains; the same method was used but utilized the RPMI media instead of Mueller–Hinton broth. The incubation period lasted for approximately 18–24 h for those plates inoculated with the test bacterial strains and for about 48 h for those plates inoculated with the *Candida* strain. The lowest concentration of *H. lanuginosum* extracts with no visible microbial growth was considered to be the MIC. The *H. lanuginosum* extracts’ antimicrobial activity was assessed in triplicate. For bacteria and fungi tested in the current work, two antibiotics and one antifungal were used as positive controls [32].

### 4.8. Cytotoxicity Analyzes

Breast cancer (MCF-7), hepatocellular carcinoma (Hep 3B and Hep G2), and cervical adenocarcinoma (HeLa) in addition to the normal human cell line (Hek293T) were obtained from American Type Culture Collection, Manassas, VA, USA (ATCC codes of HTB-22, HB-8064, HB-8065, CRM-CCL-2, CRL-3216, respectively). The cells were cultivated in an RPMI-1640 medium containing 10% fetal bovine serum, 1% streptomycin/penicillin antibiotics, and 1% l-glutamine. Cells were grown at 37 °C in a humidified environment containing 5% CO_2_. In a 96-well plate, screened cells were seeded at a density of 2.6 × 10^4^ cells per well. Following 48 h and all screened cancer and normal cells were cultured for 24 h with varying doses of the tested materials. Cell viability was determined according to the manufacturer’s directions by employing the CellTilter 96^®^ Aqueous One Solution Cell Proliferation (MTS) Assay (Promega Corporation, Madison, WI, USA). At the end of the treatment, each well was received 20 µL of MTS solution per 100 µL of medium and was incubated at 37 °C for 2 h. At 490 nm, absorbance was determined for all samples [33].

### 4.9. Free Radical Scavenging Property

The DPPH free radical scavenging activity of the *H. lanuginosum* four extracts and Trolox was performed following the method of [29]. A stock solution of a concentration of 1 mg/mL in methanol was prepared for the plant extract and Trolox. The working solutions of the following (2, 5, 10, 20, 50, and 100 μg/mL) concentrations were prepared by serial dilution with methanol from the stock solution. DPPH was freshly prepared at a concentration of 0.002% *w*/*v*. The DPPH solution was mixed with methanol and the above-prepared working concentration in a ratio of 1:1:1, respectively. The spectrophotometer was zeroed using methanol as a blank solution. The first solution of the series concentration was DPPH with methanol only. The solutions were incubated in the dark for 30 min at room temperature before the absorbance readings were recorded at 517 nm. The DPPH inhibitory percentages of the plant extracts and the Trolox standard were calculated using the following formula:

DPPH inhibition (%) = (A − B)/A × 100%

where: A = Absorbance of the blank, B = Absorbance of the sample.

The antioxidant half-maximal inhibitory concentration (IC_50_) for the plant samples and the positive control (Trolox) were calculated using BioDataFit edition 1.02 (data fit for biologist) https://www.changbioscience.com/stat/ec50.html (accessed on 1 June 2021).

### 4.10. Statistical Analyses

The statistical analyses were carried out using the SPSS software (Version 11.5, SPSS Inc., Chicago, IL, USA). All of the measurements were carried out in triplicate. The results were represented as mean values ± SDs. *p* < 0.05 was considered to be significant.

## 5. Conclusions

The quantitative phytochemical analysis of *H. lanuginosum* aqueous and methanolic extracts revealed that these extracts are rich in flavonoids and phenols. Moreover, these extracts demonstrated remarkable antibacterial and antifungal activities, especially against MRSA and *C. albicans*. The antimicrobial results showed that the aqueous extract has more potent activity against these two species, even more than the positive control antibiotics. In addition, *H. lanuginosum* polar extracts exhibited potential cytotoxic activity. All these outcomes make *H. lanuginosum* aqueous and methanol extracts good choices for developing novel antimicrobial, antioxidant, and antitumor medications. For these reasons, the present study has provided initial data that will ensure the importance of *H. lanuginosum* in conventional and traditional medicines.

## Figures and Tables

**Table 1:** Identification tests results of *H. lanuginosum* extracts.

Phytochemical Compounds	Water Extract	Methanol Extract	Acetone Extract	Hexane Extract
Flavonoid	+	+	+	-
Tannin	+	+	+	-
Phenols	+	+	+	-
Alkaloids	-	-	-	-
Saponin glycoside	-	-	-	-
Protein	+	-	-	-
Cardiac glycosides	+	+	-	-
Starch	-	-	-	-
Monosaccharides	-	+	-	-
Reducing sugar	-	+	-	-
Steroids	+	+	-	+

+ present, - absent

**Table 2:** The total phenol, flavonoid, and tannin contents of the *H. lanuginosum* extracts.

Samples	Total Flavonoid Contents, mg of QUE/g of Plant Extract, ± SD	Total Phenol Contents, mg of GAE/g of Plant Extract, ± SD	Total Tannin Contents, mg of CAE/g of Plant Extract, ± SD
Aqueous extract	76.7 ± 2.01	92.41 ± 1.54	15.11 ± 0.71
Methanol extract	28.21 ± 1.02	32.82 ± 0.81	2.61 ± 0.04
Acetone extract	0.87 ± 0.01	1.41 ± 0.25	0.87 ± 0.07
Hexane extract	-	-	-

(-): Not detected.

**Table 3:** Antimicrobial activity MIC values (µg/mL) of methanol, aqueous, hexane, and acetone extracts of *H. lanuginosum*.

Samples	Bacterium						Fungi	
	*S.* *aureus*	*E. coli*	*P.* *aeruginosa*	*S.* *sonnie*	MRSA	*E.* *faecium*	*C.* *albicans*	*E. floccosum*
Aqueous extract	0.78 ±0.01	3.13 ± 0.04	1.56 ± 0.02	1.56 ± 0.01	0.78 ± 0.01	0.78 ± 0.01	0.78 ± 0.01	0.78 ± 0.01
Methanol extract	1.56 ± 0.21	6.25 ± 0.88	3.13 ± 0.01	12.5 ± 0.41	3.13 ± 0.05	3.13 ± 0.01	1.56 ± 0.02	1.56 ± 0.02
Acetone extract	6.25 ± 0.31	12. 5 ± 0.61	12.5 ± 0.51	3.13 ± 0.31	ND	12.5 ± 0.57	3.13 ± 0.11	12.5 ± 0.15
Hexane extract	3.13 ± 0.12	ND	ND	ND	ND	ND	6.25 ± 0.11	ND
Amoxicillin	12.5 ± 0.54	25 ± 1.11	25 ± 1.01	ND	ND	12.5 ± 0.21	ND	ND
Ofloxacin	0.39 ± 0.01	0.78 ± 0.02	0.78 ± 0.02	0.39 ± 0.01	1.56 ± 0.03	0.39 ± 0.01	ND	ND
Ketoconazole	ND	ND	ND	ND	ND	ND	0.78 ± 0.01	1.56 ± 0.01

ND: Not detected.

**Table 4:** Cytotoxic activity IC_50_ values (µg/mL) of *H. lanuginosum* methanol, aqueous, hexane, and acetone extracts compared with the positive control chemotherapy drug doxorubicin.

Samples	Hep 3B	HeLa	MCF7	Hep G2	Hek293t
Aqueous extract	46.9 ± 1.01	34.11 ± 0.95	34.03 ± 0.71	85.02 ± 0.52	190.16 ± 2.67
Hexane extract	93.01 ± 2.11	54.9 ± 0.98	50.01 ± 0.87	73 ± 0.57	176.02 ± 1.99
Acetone extract	71.01 ± 1.33	75.03 ± 2.01	52.02 ± 0.39	187.91 ± 0.97	288.96 ± 1.55
Methanol extract	73.05 ± 2.51	16.07 ± 0.25	33.92 ± 0.91	182.04 ± 1.11	331.87 ± 3.12
Doxorubicin	0.99 ± 0.01	1.49 ± 0.04	1.33 ± 0.01	1.79 ± 0.11	0.58 ± 0.07

The IC_50_ was determined by MTS and is shown as mean ± SD.

**Table 5:** DPPH free radical scavenging activity of *H. lanuginosum* extracts and Trolox.

Samples	DPPH Free Radical Scavenging Activity IC_50_ Doses (µg/mL), ±SD
Aqueous extract	6.16 ± 0.75
Methanol extract	52.48 ± 1.31
Acetone extract	165.95 ± 2.31
Hexane extract	1258 ± 4.02
Trolox	2.23 ± 0.57

## Data Availability

All data is contained within the article.

## Supplementary Materials

The following supporting information can be downloaded at: https://www.mdpi.com/article/10.3390/molecules27144574/s1, Figure S1. Gallic acid standard calibration curve; Figure S2. Catechin standard calibration curve; Figure S3. Quercetin reference calibration curve; Figure S4. In vitro free radical scavenging activity of *H. lanuginosum* extracts and Trolox.

## Sample Availability

Sample of the *Hypericum lanuginosum* are available from the author.
