# Supplementary material for: Qualitative, Quantitative, Cytotoxic, Free Radical Scavenging, and Antimicrobial Characteristics of Hypericum lanuginosum from Palestine
Source: Molecules. 2022 Jul 18;27(14):4574. doi: 10.3390/molecules27144574 (PMC9323161; doi:10.3390/molecules27144574)
Supplement: Supplementary file 1 [file molecules-27-04574-s001.zip › molecules-1800635-supplementary.pdf]

Qualitative, Quantitative, Cytotoxic, Free Radical Scavenging, and Antimicrobial Characteristics of *Hypericum lanuginosum* from Palestine

Nidal Jaradat

Department of Pharmacy, Faculty of Medicine and Health Sciences, An-Najah National University, Nablus P.O. Box 7, Palestine; nidaljaradat@najah.edu

Supplementary material

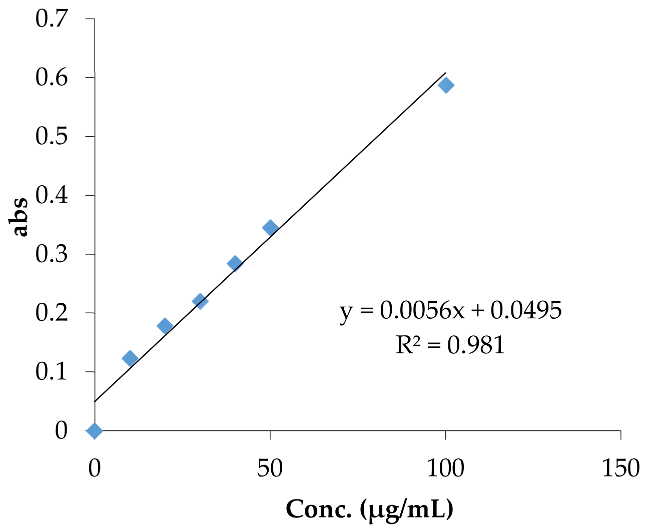

Figure S1. Gallic acid standard calibration curve

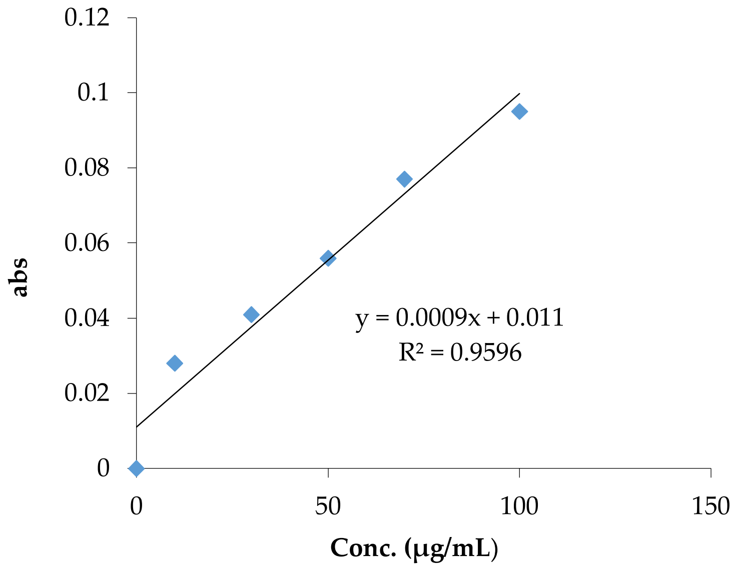

Figure S2. Catechin standard calibration curve

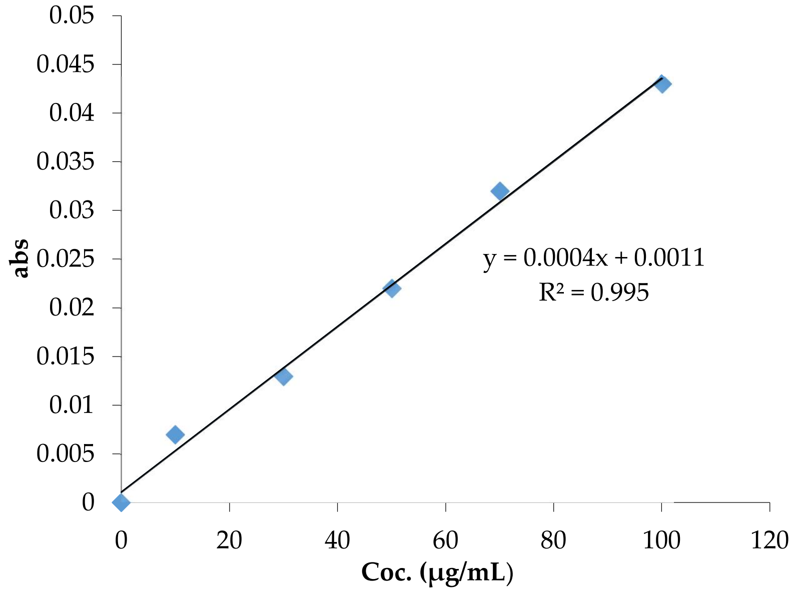

Figure S3. Quercetin reference calibration curve

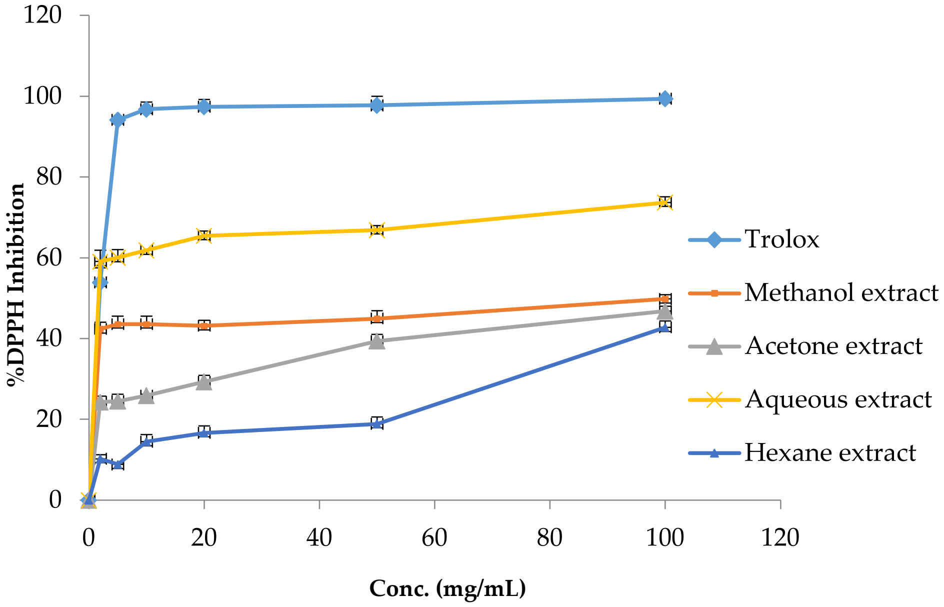

Figure S4. In vitro free radical scavenging activity of *H. lanuginosum* extracts and Trolox
